# The *pypadf* package: computing the pair angle distribution function from fluctuation scattering data

**DOI:** 10.1107/S1600576724002796

**Published:** 2024-05-17

**Authors:** Andrew V. Martin, Patrick Adams, Jack Binns

**Affiliations:** a School of Science, College of STEM, RMIT University, Melbourne, Victoria 3000, Australia; DESY, Hamburg, Germany

**Keywords:** fluctuation X-ray scattering, pair angle distribution function, X-ray cross-correlation analysis, fluctuation electron microscopy, software

## Abstract

The pair angle distribution function (PADF) is a three- and four-atom correlation function that characterizes the local angular structure of disordered materials, particles or nanocrystalline materials. The PADF can be measured using X-ray or electron fluctuation diffraction data, which can be collected by scanning or flowing a structurally disordered sample through a focused beam. It is a natural generalization of established pair distribution methods, which do not provide angular information. The software package *pypadf* provides tools to calculate the PADF from fluctuation diffraction data. The package includes tools for calculating the intensity correlation function, which is a necessary step in the PADF calculation and also the basis for other fluctuation scattering analysis techniques.

## Abstract 2

A Python-based software package is presented that can compute the pair angle distribution function from X-ray or electron fluctuation scattering data of disordered materials.

## 1. Introduction

Fluctuation scattering techniques have been developed for studying the structure of disordered materials such as colloidal materials, liquid crystals and amorphous solids (Treacy *et al.*, 2005 ▸; Kurta *et al.*, 2016 ▸; Zaluzhnyy *et al.*, 2019 ▸) and for single-particle imaging of, for example, proteins, viruses and nanoparticles (Kirian, 2012 ▸; Donatelli *et al.*, 2015 ▸; Kurta *et al.*, 2017 ▸; Pande *et al.*, 2018 ▸). Depending on the context in which they were developed, these techniques have a variety of names including fluctuation electron microscopy (Treacy *et al.*, 2005 ▸), fluctuation X-ray microscopy (Fan *et al.*, 2005 ▸), fluctuation scattering (Kam, 1977 ▸; Kam *et al.*, 1981 ▸; Saldin *et al.*, 2009 ▸, 2010 ▸) and X-ray cross-correlation analysis (Wochner *et al.*, 2009 ▸; Kurta *et al.*, 2016 ▸; Zaluzhnyy *et al.*, 2019 ▸; Lehmkühler *et al.*, 2014 ▸). These methods all use statistical approaches to extract structural information from a set of diffraction measurements (10^2^–10^5^ patterns) where the sample structure and/or orientation varies randomly between measurements. Historically, the applications to imaging and to disordered materials have developed in parallel, because the type of structural information extracted is different. In the imaging applications, the goal is to recover a 3D image of a reproducible particle, whereas for disordered materials the goal is to probe some characteristic local angular structure.

In applications to disordered materials, the sample structure is assumed to lack long-range order so that moving the beam to a new area of the sample generates statistical fluctuations in the scattering (Treacy *et al.*, 2005 ▸; Fan *et al.*, 2005 ▸). The statistical properties such as the variance (Treacy *et al.*, 2005 ▸) or angular symmetries (Wochner *et al.*, 2009 ▸) are then obtained from the ensemble of diffraction measurements. Many fluctuation techniques compute an angular intensity cross-correlation function, which captures both intensity variance and intensity cross-correlations as a function of angular separation (Kam, 1977 ▸; Kurta *et al.*, 2016 ▸). The correlation function is then used to distinguish structural models, identify the presence of symmetric local structures or map local structures. These methods have been applied to colloidal particles (Wochner *et al.*, 2009 ▸; Lehmkühler *et al.*, 2016 ▸; Liu *et al.*, 2017 ▸, 2022 ▸), nanoparticles (Lehmkühler *et al.*, 2018 ▸, 2019 ▸; Niozu *et al.*, 2020 ▸), (nano)crystals (Mendez *et al.*, 2016 ▸; Lapkin *et al.*, 2022 ▸), liquid crystals (Kurta *et al.*, 2013 ▸; Zaluzhnyy *et al.*, 2015 ▸; Martin *et al.*, 2020*b* ▸), texture in polycrystalline materials (Binns *et al.*, 2022 ▸) and metallic glasses (Liu *et al.*, 2013 ▸). Many of these experiments observe trends in the intensity correlations as a function of space, temperature or sample composition. Alternatively, correlation functions have been matched to models of local structure to extract information about the distribution of local structures. Despite this progress, the structural interpretation of correlation-based analysis results remains an outstanding issue for the field.

To address the challenge of obtaining interpretable local 3D structural information from a disordered sample, the pair angle distribution function (PADF) technique was developed (Martin, 2017 ▸). The PADF is a real-space three- and four-atom distribution that can be extracted from fluctuation scattering data by applying a linear transformation to an angular intensity cross-correlation function. It was developed to probe local 3D structure in bulk disordered materials. The PADF provides information about two atom-pair distances and the relative angle between the two pairs. It contains bond-angle information and other local angular structure that can be used to ‘fingerprint’ the local atomic arrangements in a disordered material. It has been applied with X-rays to identify local structures in self-assembled lipid phases (Martin *et al.*, 2020*b* ▸) and with electrons to study defects in disordered carbon materials (Martin *et al.*, 2020*a* ▸), and there are prospects for studying proteins (Adams *et al.*, 2020 ▸) and close-packed colloidal particles (Bøjesen *et al.*, 2020 ▸). The PADF is primarily designed for fluctuation studies of disordered materials because these are the cases where it remains difficult to obtain structural insights from the analysis of the correlation function in *q* space. In principle, the PADF could be calculated from single-particle fluctuation data, but it does not provide a 3D image of the sample unlike other approaches (Donatelli *et al.*, 2015 ▸).

The PADF is different from the 3D-ΔPDF (Schmidt *et al.*, 2023 ▸), which can be used to map the pair displacements of defects in microcrystals with the 3D orientation resolved. The 3D-ΔPDF technique requires a crystalline structure to enable crystallographic methods with the sample orientation resolved. While the PADF can be applied to crystalline materials, it does not require crystals. It is also based on the assumption that information about sample orientation is lost in the collection of serial (fluctuation) diffraction data. Hence, the 3D-ΔPDF may have advantages for defective or dis­ordered crystals, while the PADF has the advantage that it can be applied to a wider class of materials.

Here we present *pypadf*, a Python3 package for the calculation of the PADF from diffraction data. The code provides tools to (i) calculate a *q*-space correlation volume from a fluctuation scattering data set, (ii) apply masks and geometric corrections to the correlation volume, (iii) calculate the PADF from the correlation volume, and (iv) plot intensity correlation and PADF volumes.

## 2. Overview of the *pypadf* package

**Figure 1:**
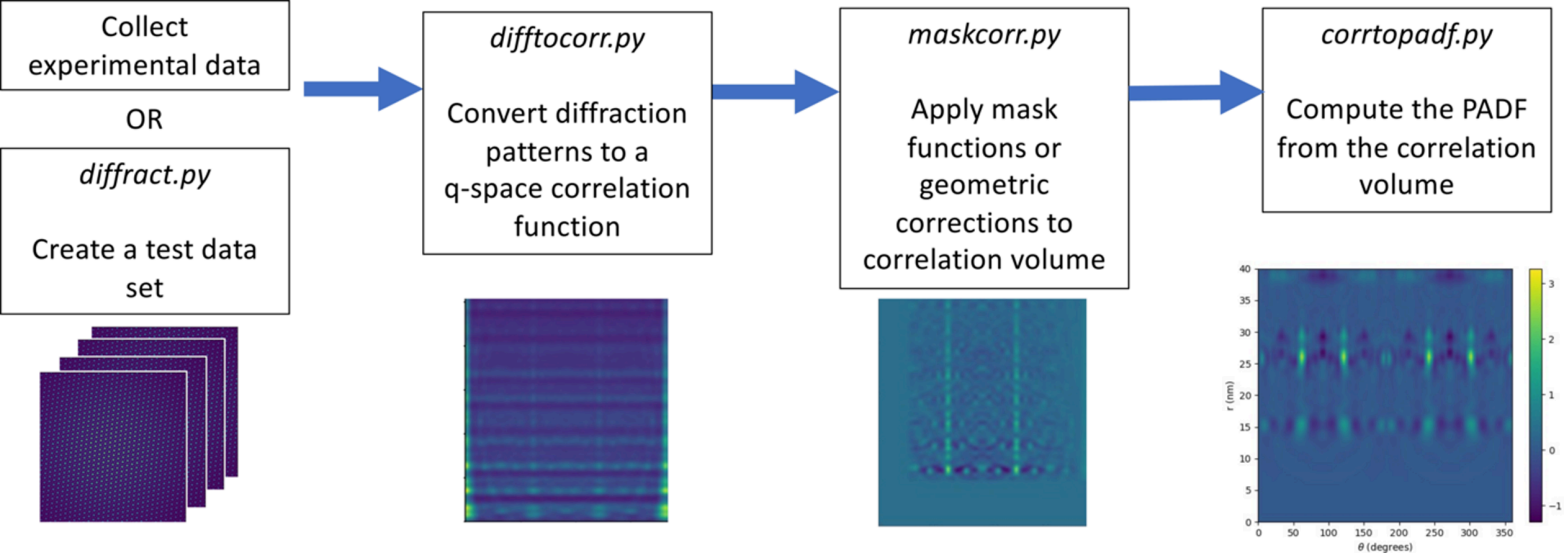
A schematic diagram of the workflow for computing the *q*-space correlation function and the PADF. The *pypadf* package consists of separate scripts that are run in the order indicated by the arrows. The images are illustrative of the output at different stages and are described in more detail in Section 3 and Fig. 3.

The *pypadf* package has three parts: (i) the main scripts, (ii) the *params* module containing all input parameter specifications, and (iii) the *fxstools* module which contains the tools for calculating, analysing and plotting correlation functions. As shown in Fig. 1 ▸, the main scripts are difftocorr.py to convert diffraction patterns to a correlation function, maskcorr.py to prepare the correlation function for the PADF calculation and corrtopadf.py which computes the PADF from the correlation function. The correlation and PADF functions can be plotted with the script plotfxs3d.py. Each script imports a submodule from the *params* module that defines parameters specific to that script. The scripts take input parameters from a configuration file or via command line options. The configuration file is read first and then command line arguments are read second. Hence, parameter values from command line arguments take precedence over (*i.e.* will override) parameter values defined in the configuration file. This enables the command line options to be used in batch scripts where a small number of parameters are changing for each data set. In the rest of this section we detail the main scripts for computing the PADF, including diffraction simulation, correlation calculation and finally the PADF calculation. For each step we outline the background theory, the numerical implementation of the equations and the script associated with each step.

The *pypadf* package includes scripts that can create test data sets called diffract.py and diffract_and_correlate.py. These scripts are summarized in Appendix *A* [App appa] and they assume elastic scattering and no absorption. We note that, for detailed simulation studies, there are established diffraction programs available for both single particles and crystals, such as *Reborn* (Kirian *et al.*, 2020 ▸; Chen *et al.*, 2021 ▸), *Condor* (Hantke *et al.*, 2016 ▸) and *MLFSOM* (Holton *et al.*, 2014 ▸).

### 2.1. The angular intensity correlation function *C*(*q*, *q*′, θ)

#### 2.1.1. Intensity correlations: mathematical and numerical details

The angular intensity correlation function is calculated from the polar representations of the diffraction data *I*(*q*, θ), where *q* is the vector magnitude of **q** (defined in Appendix *A* [App appa]) and θ is the angle around the beam axis: 

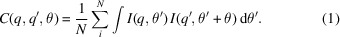


*N* is the number of diffraction patterns in the data set. We assume that the diffraction patterns have been corrected for any solid-angle and polarization effects. The corrections are not currently implemented in difftocorr.py. In the polar representation, each *q* and *q*′ value labels an intensity ring. Numerically, the 1D fast Fourier transform and the convolution theorem are used to compute the angular correlation between each pair of rings *q* and *q*′.

In principle, equation (1) can be computed for any set of diffraction patterns, but to make *C*(*q*, *q*′, θ) suitable for PADF analysis there are some extra requirements. We assume that the local structures in a disordered material (or particles) have no preferred orientation with respect to the beam axis. Preferred orientation effects have been observed in PADF experiments. The sizes of these effects depend on the beam size (Binns *et al.*, 2022 ▸), and they were identified because the angular peaks in the PADF had no sensible nanostructural interpretation.

The number of diffraction patterns required for *C*(*q*, *q*′, θ) to converge depends on the ratio of the beam size to the length scale of the order in the sample (or particle size), and the lowest number of patterns will be required if the beam can be focused close to the structural correlation length in the sample (or particle size). The number of patterns also depends on the resolution and the beam intensity. Typically for high signal-to-noise data sets, it has been found in experiments to date that the order of 10^3^ or 10^4^ patterns will be required. Theoretically it may be possible to measure weaker signals with XFELs using 10^6^ or 10^7^ patterns (Martin, 2017 ▸). It is not usually possible to estimate the precise number of patterns required in advance, and convergence is checked by comparing *C*(*q*, *q*′, θ) computed from independent subsets of the data.

By default, the script difftocorr.py computes the correlations from all odd-numbered and even-numbered frames independently and outputs them as the ‘a’ and ‘b’ correlation functions. The PADFs computed from the ‘a’ and ‘b’ correlation results can be compared visually to detect changes due to incomplete convergence (or alternatively *q*-space correlation functions can be compared visually instead). This convergence check is appropriate when the beam size is smaller than the distance between neighbouring measurements, so that the sample volumes in neighbouring measurements contain no common atoms. If neighbouring probe positions overlap, the ‘a’ and ‘b’ results will not be independent and will not give a reliable indication of convergence.

In the *pypadf* package, the magnitude of the vector **q**_*i*_ associated with the *i*th pixel is defined to be 

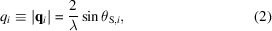

where the scattering angle of the *i*th pixel is defined as 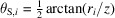 , *r*_*i*_ is the radial distance of the pixel centre from the beam centre and *z* is the sample-to-detector distance. We note that the definition of *q*_*i*_ uses a convention common in electron scattering applications and differs from the usual convention in X-ray diffraction by a factor of 2π. In X-ray sciences *q*_X-ray_ = 2π|**q**_*i*_| = 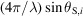 .

A consequence of Ewald sphere curvature, expressed by equation (2), is that pixels of a uniform width do not generate uniform sampling of *q*_*i*_ ≡ |**q**_*i*_|. The diffraction data are interpolated onto a uniform sampling of *q* when it is mapped onto polar coordinates *I*(*q*, θ). The interpolation is implemented using the *map_coordinates* function from the *scipy.ndimage* module (Virtanen *et al.*, 2020 ▸).

The correlation background due to static signals can be estimated from the cross-correlation of independent diffraction patterns. Such static signals include background scattering in the measured images that does not vary from frame to frame. We note that this estimate is only valid for samples with uniformly random orientations. If there are preferred orientations then the cross-correlations will also include some of the useful signal from the sample. The effect of static background signals can be estimated by randomly correlating pairs of diffraction patterns, 

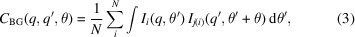

where *j*(*i*) is a randomly chosen index that is not equal to *i*. This background estimate can be subtracted from the estimate of the correlation signal made by equation (1).

A background-subtracted correlation function can be computed in a single pass over the data by computing difference correlations (Mendez *et al.*, 2016 ▸), 

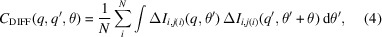

where Δ*I*_*i*,*j*(*i*)_(*q*, θ′) = *I*_*i*_(*q*, θ′) − *I*_*j*(*i*)_(*q*′, θ′) for pairs of randomly picked diffraction patterns *j*(*i*) ≠ *i*. It can be shown that *C*_DIFF_ = 2(*C* − *C*_BG_).

A mask can be applied to exclude the beamstop, detector gaps and bad pixels from the analysis. In the *pypadf* package, a binary mask is used that takes a value of 1 for included pixels and 0 for excluded pixels. The effect of the mask on the correlation function is corrected by dividing the correlation function by the correlation of the mask: *C*_corrected_(*q*, *q*′, θ) = *C*(*q*, *q*′, θ)/*C*_mask_(*q*, *q*′, θ) wherever *C*_mask_(*q*, *q*′, θ) > 0. In places where the correlation of the mask is 0, the corrected correlation function is set to zero.

#### 2.1.2. difftocorr.py: computing correlations from diffraction patterns

The script difftocorr.py computes a correlation function from a set of diffraction patterns. It can compute *C*(*q*, *q*′, θ), *C*_BG_(*q*, *q*′, θ) or *C*_DIFF_(*q*, *q*′, θ) and it can perform the mask correction. By default it calculates two correlation functions from the odd and even frames, which can be compared to check visually that the correlation functions have converged. The comparison of odd- and even-frame correlations is appropriate if the subsequent measurements are taken from statistically independent sample regions. If they are not independent, then different subsets of the data may need to be compared.

The script assumes that diffraction patterns are saved in individual files and it constructs a file list from the folder based on a filename format specified in the configuration file. There are parameters to centre, crop, rebin and mask the diffraction patterns. The processed diffraction patterns can be saved to check that the processing parameters are correct. The script requires detector geometry parameters including the sample-to-detector distance, the beam centre, the width of a detector pixel and the wavelength. The output of the script is the 3D correlation function saved in a *NumPy* file or as a raw binary file.

#### 2.1.3. maskcorr.py: applying corrections to the correlation volume

The script maskcorr.py makes modifications to the correlation volume prior to computing the PADF. Accurate calculation of the PADF requires the correlation volume to be evenly sampled with respect to 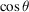 due to the orthogonality conditions of the Legendre polynomials. However, the correlation function is most conveniently calculated with uniform θ sampling. The maskcorr.py script can multiply the correlation function by 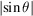 to correct for this sampling effect.

In an experiment, it can occur that the effects of background scattering or other artefacts are still evident after calculating *C*_DIFF_ or subtracting *C*_BG_. If these spurious signals are confined to a particular region of the correlation function, such as low *q* values, they can be masked. The script maskcorr.py can apply low- and high-pass filters on the *q* dimensions.

Noise on the diffraction pattern causes a peak at θ = 0 and *q* = *q*′, which is equal to the variance of the noise in each *q* ring. Assuming that the noise is uncorrelated on different pixels on the detector, then the spurious noise correlations are confined near θ = 0 and do not affect the rest of the correlation function. A mask can be applied to the region close to θ = 0, which can remove this noise-variance signal. The origin of the noise may be detector noise or noise from spurious correlations in the sample, *e.g.* across distances larger than the structural correlation length in the sample or coherent interference between distant atoms.

### 2.2. The pair angle distribution function Θ(*r*, *r*′, θ)

#### 2.2.1. The PADF: mathematical and numerical details

Here we summarize the theory and numerical implementation of the transformation of the correlation function *C*(*q*, *q*′, θ) into the pair angle distribution functio Θ(*r*, *r*′, θ).

The modulus squared of the sample’s scattering factor can be expanded in terms of spherical harmonics as

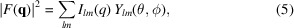

where *Y*_*lm*_(θ, ϕ) are spherical harmonic functions. We assume that measurements of |*F*(**q**)|^2^ on the Ewald sphere are accessible experimentally via kinematic scattering, which is appropriate for high-energy X-rays that scatter weakly. Electrons scatter more strongly than X-rays and exhibit dynamic scattering, which impacts quantitative peak-height analysis of pair distribution functions (Anstis *et al.*, 1988 ▸). It remains to be verified whether this has a similar effect on the PADF.

It can be shown that the correlation function has the form 

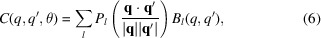

where *P*_*l*_(*x*) are the Legendre polynomials. The *B*_*l*_(*q*, *q*′) matrices are given by 

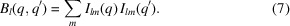

The *B*_*l*_(*q*, *q*′) matrices can be extracted by numerically inverting equation (6) using singular value decomposition or by using the orthogonality properties of the Legendre polynomials. The default behaviour in the *pypadf* package is to use singular value decomposition. A value of 0.5 is used to regularize the small singular values, which was selected to exclude singular values near to 0, but it can be changed by the user. The singular values depend on the experimental geometry, via the Ewald sphere, but do not depend on the input data. The value of 0.5 has been found to be adequate for all applications and tests that we have made to date.

The *B*_*l*_(*q*, *q*′) terms are converted to real space using two spherical Bessel transforms, 

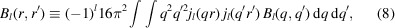

where *j*_*l*_(*x*) denotes a spherical Bessel function of order *l*. The spherical Bessel transform is implemented using the discrete form of Lanusse *et al.* (2012 ▸). In the discrete form, a general function *f*_*l*_(*q*) of order *l* and radial coordinate *q* is transformed to a real-space function by 

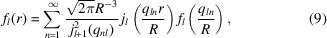

where *R* is the maximum value of *r* and *q*_*ln*_ is the *n*th zero of the *l*th spherical Bessel function. To implement this, all *B*_*l*_(*q*, *q*′) matrices are first computed on the zero positions of the *l* = 0 spherical Bessel function. Interpolation is then used to remap matrices with *l* > 0 onto the appropriate *q* sampling points before using equation (9) to compute the real-space *B*_*l*_(*r*, *r*′) matrices (Lanusse *et al.*, 2012 ▸).

The code computes a real-space correlation function by forming a weighted sum of the *B*_*l*_(*r*, *r*′) matrices:

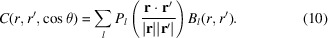

The function 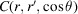 is a scaled form of the pair angle distribution function Θ(*r*, *r*′, θ) as follows: 

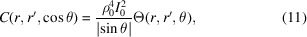

where 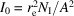 , recalling that solid-angle and polarization effects are already assumed to be corrected. Here ρ_0_ is the mean density in the sample and *N*_I_ is the number of incident photons (electrons) in the exposure. The 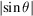 term corrects for a factor that arises in the derivation of the PADF using spherical coordinates (Martin, 2017 ▸).

It can be shown that the PADF can be written as 

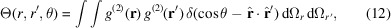

where *g* ^(2)^(**r**) is the two-atom distribution function in 3D, 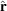 and 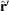 are unit vectors and dΩ_*r*_ is the solid-angle element associated with the coordinate **r**. Equivalently, the PADF can be written as

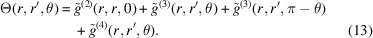

**Figure 2:**
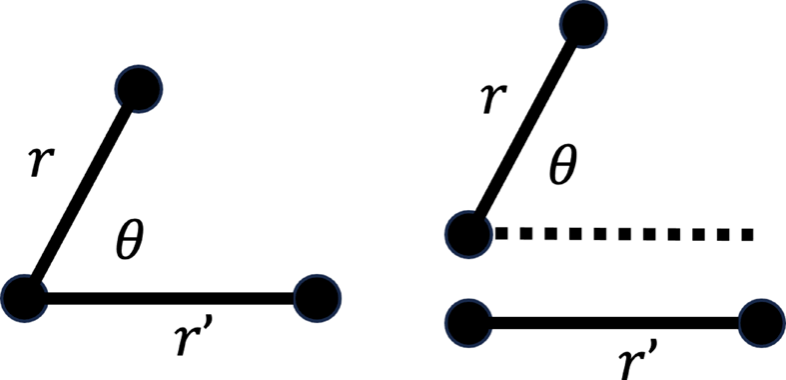
The relevant coordinates of (left) three-atom combinations and (right) four-atom combinations that contribute to the PADF. The PADF is not sensitive to absolute position, absolute orientation or, in the four-atom case, the separation distance between the two atom pairs.

The functions 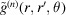 are multi-atom correlation functions, parametrized by two pair distances and a relative local angle. Note the *n* = 2 term is only non-zero where *r* = *r*′. The tilde symbol indicates that these terms differ from the general correlation functions of statistical mechanics by integrating out the degrees of freedom that the diffraction is insensitive to, such as the absolute position and absolute orientation of the pairs and the distance between the pairs. The remaining degrees of freedom are shown in the diagram in Fig. 2 ▸. Further detail about the definitions of these functions can be found in the report by Martin (2017 ▸).

#### 2.2.2. corrtopadf.py: computing the PADF from the correlation function

The script corrtopadf.py converts the *q*-space correlation function into the PADF. It calculates the *B*_*l*_(*q*, *q*′) matrices, applies the numerical spherical Bessel transforms to obtain *B*_*l*_(*r*, *r*′) matrices and then reconstructs the PADF. The *B*_*l*_(*q*, *q*′) and *B*_*l*_(*r*, *r*′) matrices can be saved as optional output. The number of spherical harmonics is set to control the angular resolution and only even spherical harmonics are used, because the inclusion of odd harmonics reduces the accuracy of the matrix inversion. The approximation to remove odd harmonics is valid when absorption is neglected. As per equation (11), the output 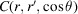 can be multiplied by 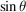 to produce a function proportional to the PADF. There is an option to multiply by the constants in equation (11) to obtain absolute values of the PADF.

We note that the current version of the *pypadf* code has been developed further since the first experimental demonstrations (Martin *et al.*, 2020*a* ▸,*b* ▸; Adams *et al.*, 2020 ▸). The numerical accuracy has been improved via changes to the normalization of basis functions and the inclusion of 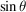 terms that arise from angular sampling considerations. These improvements produce more accurate peak heights in the PADFs. The structural interpretations of the early experimental papers were primarily based on the angular peak positions, which are less affected by these improvements to the code. Hence, we consider that the conclusions of the first PADF studies are still valid.

## 3. An example PADF calculation

**Figure 3:**
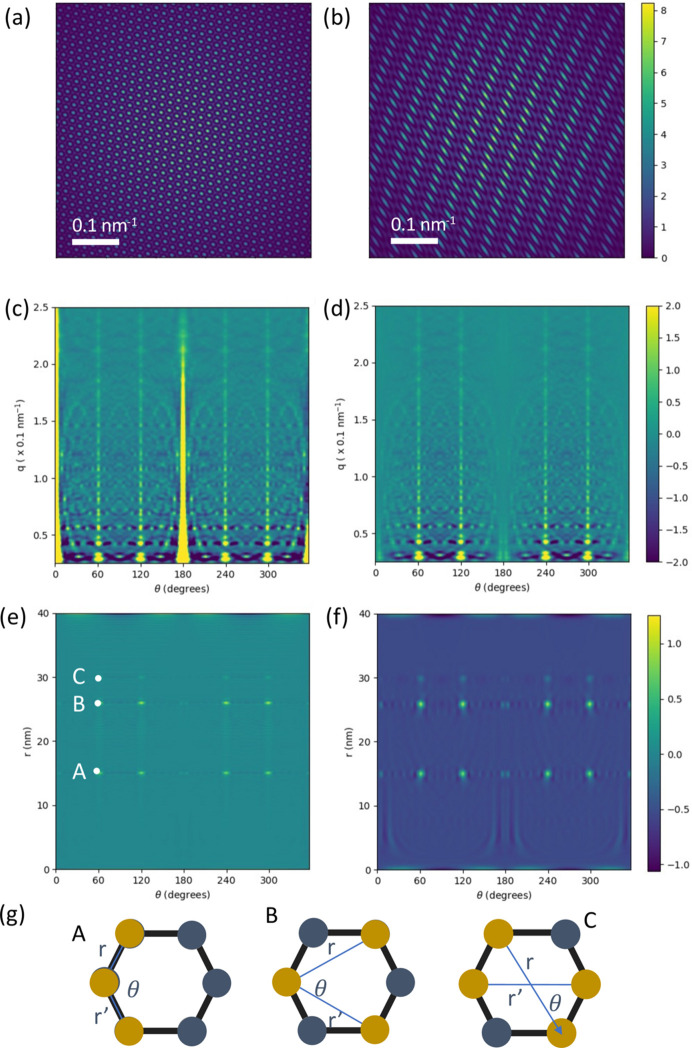
(*a*), (*b*) Two diffraction patterns from the model hexagonal sample. (*c*) The *q*-space correlation function for the hexagonal model calculated from 1000 patterns without the 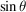 correction. (*d*) The *q*-space correlation function from panel (*c*) with the 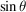 correction. (*e*) The PADF of the hexagonal structure computed from the corrected *q*-space correlation function. (*f*) The PADF convolved with a narrow Gaussian kernel. (*g*) The pair combinations that generate the points A, B and C that are marked in panel (*e*).

Here we provide an example of a PADF calculated from a simulated set of fluctuation scattering diffraction patterns. The model sample contains six point scatterers in a hexagonal arrangement with a nearest-neighbour distance of 15 nm. For each pattern the diffraction pattern was rotated to a random orientation. A data set of 1000 diffraction patterns was simulated with diffract.py on a 512 × 512 pixel grid. The maximum *q* value recorded at the edge of the detector is 1.28 nm^−1^, which corresponds to a resolution of 0.78 nm. Example diffraction patterns from the data set are shown in Figs. 3 ▸ *(a*) and 3 ▸(*b*). The regularity of the observed interference patterns arises from the geometric arrangement of the six scatterers and their absolute orientation. There is a weak attenuation at high *q* values due to the atomic scattering factor and the reduced solid angle of pixels near the edge of the detector. No noise is modelled on the detector.

The correlation function computed from all 1000 simulated patterns is shown in Fig. 3 ▸(*c*). Since no background signals have been modelled, the standard correlation function *C*(*q*, *q*′, θ) defined by equation (1) has been calculated. The highest *l* value in the spherical harmonic expansion was 32, which corresponds to an angular resolution of 11.25°.

There are strong features at angles of 60° and 120°, which are expected from the hexagonal arrangement of atoms in the sample. The correlation function is strongest at 0° and 180°, which is expected because it has been calculated with a regular sampling of θ. Fig. 3 ▸(*d*) shows the correlation function after applying a 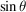 scaling with maskcorr.py (see Section 2.1.3) as this is a necessary prior step for accurate PADF measurements. The PADF was calculated using corrtopadf.py and the result shows the expected angular peaks for a hexagonal arrangement of scatterers at *r* = *r*′ = 15, 26 and 30 nm and at 60° and 120° [see peaks labelled A, B and C in Fig. 3 ▸(*e*)]. There are some weaker oscillatory artefacts that are caused by the finite radial and angular sampling. Fig. 3 ▸(*f*) shows the result on convolving (*e*) with a small Gaussian kernel (radial half-width of 0.75 nm and angular half-width of 1°), which reduces the pixelization effects at the peak location and assists in identifying peak heights. The ratios of the peak heights of the convolved PADF are within 11% of the ideal peak ratio values, as shown in Table 1 ▸. This shows that both the peak positions and the peak heights can be analysed quantitatively.

We would expect results approaching this accuracy to be obtainable from X-ray experiments, if the sample and experimental conditions can be modelled by kinematic scattering and no absorption. For electron diffraction, it is not yet known how much additional error may arise from dynamic scattering.

As mentioned above, the raw output of corrtopadf.py is multiplied by 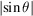 to generate the PADF, and this is necessary for analysing peak heights. However this multiplication is inconsistent with the finite number of angular basis functions used and artificially lowers the values near θ = 0. Hence, peak heights near θ = 0 cannot be analysed quantitatively yet. The range of affected angles around 0 depends on the angular resolution, as defined by the number of spherical harmonics used, which for our example is about ±6°. A modified form of the multiplicative 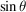 term is needed, but a numerically reliable modification is not yet known.

The widths of the peaks are determined by the finite radial and angular resolution. The radial resolution is set by the maximum *q* value used in the calculation of the correlation function, which is set to be the *q*-space distance from the centre of the diffraction pattern to the detector edge. The angular resolution of the PADF is set by the maximum value of *l* used in the calculation, which was *l*_max_ = 30.

There is angular structure that is not expected from the ideal structure, which is weaker than the principal angular peaks. These are artefacts created by truncating the basis sets used, which are analogous to Fourier artefacts in signal processes caused by the truncation of the Fourier series expansion. The truncation artefacts can be reduced by increasing the angular and radial resolution, but cannot be removed entirely because the experimental data converge at a finite resolution.

Aside from the *r* = *r*′ slices shown in Fig. 3 ▸, the plotfxs3d.py script can plot other 1D and 2D sections from the 3D PADF volume. Current options include a 2D slice of constant θ value or constant *r* value, and 1D radial or angular lines.

## 4. Access to *pypadf*

The *pypadf* package can be downloaded from https://github.com/amartinrmit/pypadf and is distributed under the GNU Lesser General Public Licence (LGPL, Version 3; https://www.gnu.org/licenses/lgpl-3.0). The *pypadf* package is written in Python3 and requires the following packages: *NumPy*, *SciPy*, *MatPlotLib*, *Numba*, the Python imaging library (PIL) and *h5py*. The configuration and input files for the hexagonal example shown here are included with the code. The readme. md file contains instructions for installation, and a list of possible parameters for each script can be found with the -help command line argument.

## 5. Conclusion and future work

We have presented the *pypadf* package, which can compute the pair angle distribution function from fluctuation scattering diffraction data. The package includes scripts that can simulate diffraction patterns, compute angular correlation functions, modify angular correlation functions, compute the PADF and finally plot the results. The analysis assumes kinematic scattering approximations, no absorption, and that each diffraction pattern is of a sample in a random orientation or a statistically independent region of a bulk disordered sample. We expect the code to be useful for probing local 3D structures in disordered materials probed with X-ray and electron beams.

Scanning and serial diffraction experiments are well established data collection methods with electron microscopes, synchrotrons and X-ray free-electron laser facilities. We expect that many existing fluctuation data sets are suitable for PADF analysis and that many facilities already have the capability of measuring these data sets.

Further work is still required to understand the convergence of the correlation functions and how to reduce numerical artefacts in the linear transformations, interpolations and matrix inversions that are used. For electron diffraction calculations, the effect of dynamic diffraction is yet to be investigated.

## Figures and Tables

**Table 1:** Ratios of the peak heights recovered from the diffraction simulation The labels A, B and C are defined in Fig. 3 ▸(*e*). *R*_A/B_ stands for the ratio of the height of peak A to the height of peak B, and *R*_C/A_ denotes the ratio of the height of peak A to the height of peak C.

Peak ratio	Ideal value	Recovered value
*R*_B/A_	1	1.11
*R*_C/A_	0.25	0.26

## Appendix A Diffraction scripts

### A1. The input data: far-field diffraction patterns

#### A1.1. Diffraction: mathematical and numerical details

The input data for the correlation calculation are a set of diffraction patterns *I*(**q**), where **q** = **q**_s_ − **q**_0_ is the difference between the scattering **q**_s_ and incident **q**_0_ wavevectors. The kinematic diffraction approximation (single elastic scattering) is assumed. The intensity for a uniform incident pulse is given by

where *r*_e_ is the classical electron radius, *P*(**q**) is a polarization factor, dΩ is the solid angle of a pixel, *A* is the beam area, *N*_I_ is the number of incident photons (electrons) and *F*(**q**) is the molecular scattering factor, which is calculated from the atomic positions.

Due to the Ewald sphere, the solid angle subtended by a pixel reduces if the pixel is located further away from the beam centre. The reduction in solid angle is approximately given by  , where dΩ_0_ is the solid angle of a pixel at the beam centre and θ_S,*i*_ is the scattering angle defined in equation (2). There is an option to apply this approximation in diffraction pattern simulations and the effect becomes significant for wide-angle diffraction.

#### A1.2. diffract.py: computing test diffraction data

The script diffract.py can be used to compute basic diffraction data for testing the correlation and PADF scripts. This script computes a diffraction pattern of a randomly orientated molecule from atomic coordinates given in a Protein Data Bank file (.pdb; https://www.rcsb.org/). It does not use any unit-cell or crystal lattice information and hence only simulates continuous diffraction. The atomic scattering factors are taken from Waasmeier & Kirfel (1995 ▸), and data from Henke *et al.* (1993 ▸) are used for wavelength-dependent corrections. A square detector is assumed and its distance from the sample, pixel width and number of pixels along a side length can be varied.

#### A1.3. diffract_and_correlate.py: testing large data sets

When simulating large diffraction data sets, it can be impractical to store every diffraction pattern prior to calculating the correlation function. The script diffract_and_correlate.py simulates diffraction patterns and correlates those patterns on the fly. Only a small number of diffraction patterns are created at any one time. They are then correlated and deleted and the cycle is repeated. A single atomic structure can be used, which can be randomly rotated and translated with periodic boundary conditions to generate the diffraction patterns. This script takes the same parameters as diffract.py and difftocorr.py.
